# An Exploratory Study of the Relationship Between Phoria, Oculomotor Skills and Visual Symptoms in Children Aged 5 to 8 Years

**DOI:** 10.3390/jemr19020036

**Published:** 2026-04-02

**Authors:** Carmen Bilbao, Julia Cavero, Jorge Ares, Alba Carrera, Diana Gargallo

**Affiliations:** 1 Department of Optometry, Hospital Quirón Salud Huesca, 22004 Huesca, Spain; juliacaverovalles@gmail.com (J.C.); albass.acb@gmail.com (A.C.); 2 Department of Applied Physics, Universidad de Zaragoza, 50009 Zaragoza, Spain; fatxutxa@unizar.es (J.A.); dgargallo@unizar.es (D.G.); 3 Hospital Universitario Miguel Servet, 50009 Zaragoza, Spain

**Keywords:** eye movement, saccades, smooth pursuit, vergence

## Abstract

Purpose: To investigate the relationship between oculomotor skills, phorias, and visual symptoms in pediatric population aged 5 to 8 years. Methods: A cross-sectional study was conducted with 120 children, divided into three age groups. Each participant underwent a full optometric examination, including the Maddox test for dissociated phoria, and the Northeastern State University College of Optometry (NSUCO) and Developmental Eye Movement (DEM) tests for oculomotor function. In addition, the Convergence Insufficiency Symptom Survey (CISS V-15) questionnaire was administered to assess visual symptoms. Results: The prevalence of binocular and oculomotor dysfunctions varied by age and sex. Differences in saccadic and pursuit eye movement performance were observed between groups. Older children showed patterns of association between phoria measurements, oculomotor performance, and possible visual symptoms, particularly in girls over 6 years of age. Conclusions: This study provides additional descriptive data for the pediatric population and highlights that oculomotor dysfunction and phoria frequently coexist. Symptom scores measured by the CISS V-15 tended to increase with age. The results should be considered preliminary and potentially hypothesis-generating, pending the future availability of a validated questionnaire to assess phoria-related symptoms in children from 5 years of age. Overall, this study underscores the importance of comprehensive binocular vision assessments in school-aged children.

## 1. Introduction

Binocular vision refers to the ability of both eyes to coordinate and integrate their input to produce a single, clear, and three-dimensional image. This function depends on proper alignment, motor coordination, and sensory fusion. Binocular vision begins to develop early in childhood, with basic sensory and motor fusion typically functioning between 6 and 9 years of age, and stereoacuity threshold values reaching adult levels [1,2]. Proper development of binocular vision is crucial for reading and learning, where precise eye alignment and coordination are also necessary for efficient visual processing [3].

A dissociated phoria is a latent ocular deviation that becomes evident when binocular fusion is interrupted. Under normal visual conditions, sensory and motor fusion keep the eyes aligned; however, when fusion is eliminated, such as during a cover test or with prism dissociation, the eyes move to their resting positions, revealing the phoria. The magnitude and direction of this deviation are measured at a specific viewing distance, commonly at near or distance. Near vision phorias are primarily classified based on the direction of ocular misalignment, with a particular focus on horizontal deviations: esophoria, characterized by a latent inward deviation and associated with high tonic convergence, and exophoria, defined as a latent outward deviation linked to low tonic convergence. Phoria variability has been studied under certain environmental conditions [4]. The type of phoria observed provides insight into the patient’s binocular alignment resiliency and is essential for diagnosing vergence disorders [5].

Deficits in binocular vision, particularly those involving elevated dissociated near exophoria, such as convergence insufficiency (CI), have been associated with a range of visual symptoms, including eyestrain and blurred vision. These symptoms can adversely affect binocular vision, reducing reading speed and comprehension, which can negatively impact academic performance [6,7]. The Convergence Insufficiency Symptom Survey (CISS V-15) is a test developed to detect patients with CI [8], as well as to monitor their response to treatments such as vision therapy. Additionally, it is useful for distinguishing between symptoms related to visual problems and those caused by other conditions, such as learning or reading difficulties.

The information reported in the literature regarding the prevalence of phorias in the pediatric population is diverse, age-dependent, and influenced by the definition used. In an Australian study by Leone JF et al. [9] conducted in 6-year-old children, near exophoria was predominant (58.3%), while orthophoria predominated at distance (85.4%). Cases of esophoria were scarce at both distances (1.0% and 9.2%, respectively). In contrast, in a study of Iranian children from rural regions under 5 years of age [10], the prevalence of distance exophoria and esophoria was 4.8% and 0.3%, and the prevalence of near exophoria and esophoria was 26.0% and 0.5%, respectively. In any case, at both distances, orthophoria was predominant (73.5% for near vision and 95.0% for distance vision). Finally, in a retrospective evaluation of Canadian children aged 6 to 14 years with reading difficulties [11], orthophoria was found to be the most prevalent condition, both at distance (90%) and at near (65%). Among cases in which phorias were present, again exophoria was observed more frequently than esophoria, with prevalence rates of 8% versus 2% for distance vision, and 34% versus 1% for near vision, respectively.

Recent findings indicate that 35% of children with reading difficulties exhibit near exophoria outside of the expected ranges [11]. Learning or reading difficulties do not necessarily cause visual symptoms; however, they may share certain signs with visual impairments, such as decreased attention span, skipping lines while reading, or reduced reading speed. These similarities can lead to confusion, as the etiology is usually related to cognitive processing, rather than ocular or binocular disorders [12]. Children with exophoria may experience visual discomfort due to the increased vergence demand needed to maintain ocular alignment, suggesting a possible link between binocular vision anomalies and reading difficulties. This data highlights the importance of assessing binocular vision in pediatric populations, as phorias could affect reading acquisition and visual processing efficiency, particularly in children with exophoric tendencies [11]. Phorias play an important role in the efficiency of binocular vision, with direct implications for reading acquisition in children, demonstrating that abnormal phoria values, particularly near exophoria, are associated with blurred vision, visual fatigue, and difficulty sustaining attention during reading [13]. Palomo-Alvarez et al. [14] further reported that these visual difficulties can manifest as reduced reading speed, frequent loss of location, line skipping, or the need to reread text, all of which negatively affect fluency and comprehension, ultimately impacting academic performance.

Beyond reading acquisition, phorias have also been shown to influence basic visual processing. Studies found that heterophoria can disrupt spatial interactions in a manner similar to meridional amblyopia [15,16], even in the absence of refractive errors or overt strabismus. Their work also showed that high heterophoria increases the intensity and spatial extent of foveal crowding, which may limit visual efficiency and contribute to fatigue.

The clinical relevance of assessing phorias extends beyond reading and visual processing tasks [17], emphasizing that detecting abnormal phorias is critical for the early identification of binocular vision disorders such as convergence insufficiency, decompensated heterophoria, or amblyopia. Accurate assessment can therefore support both educational outcomes and ocular health management in pediatric populations.

Oculomotor function refers to the coordinated motor ability of the eyes to control the gaze position in response to static or moving objects [18]. Fixation is the action of holding the gaze on a specific static visual stimulus, keeping it stable on the retina [19]. Two types of ocular movements (OM) exist to shift the fixation point position: pursuits and saccades. Pursuits are slow, smooth, conjugate movements of both eyes designed to maintain foveal stability on stimuli moving within the visual field [19]. Saccades are rapid eye movements that quickly shift fixation from one point of interest to another, aligning the object of focus with the fovea on the retina [20].

Oculomotor dysfunctions are not only independent alterations in the motor control of the eyes but also frequently coexist with binocular dysfunctions, establishing a bidirectional relationship that can exacerbate visual difficulties in pediatric populations. These combined dysfunctions negatively impact the visual system’s ability to integrate and coordinate precise eye movements, affecting binocular alignment, which is a critical factor for the development of fine motor skills such as grasping objects [21].

Research has shown that the magnitude of phoria correlates with key aspects of binocular vision, including vergence response and dynamics [22,23,24,25]. Individuals with phorias often show greater variability in their fixation compared to those with proper ocular alignment [24]. These findings suggest that ocular misalignments may influence not only visual processing but also the control and precision of vergence movements, potentially affecting performance in visual tasks.

In this work, an experimental measurement study was conducted on a sample of the pediatric population. In addition to the classical visual function measurements, the study included results from the Developmental Eye Movement Test (DEM) [26], Northeastern State University College of Optometry (NSUCO) oculomotor test [27], Convergence Insufficiency Symptom Survey (CISS V-15), and Maddox tests, among others. The main aim of this study was to investigate the relationship between phorias and oculomotor skills in children aged 5 to 8 years. Additionally, we sought to explore the associations of these binocular and oculomotor measures with visual symptoms, to understand whether oculomotor or binocular dysfunctions are linked to self-reported discomfort in this population.

## 2. Methods

### 2.1. Participants

Recruitment took place at Colegio Salesianos de Huesca between September and December 2023 and included all eligible children aged 5 to 8 years from this institution. Parents provided written informed consent prior to the tests, allowing participation and data processing. The study design was approved by the ethics committee at the University of Valencia (CEIUV, 2703478, approval date 1 June 2023). The research adhered to the tenets of the Declaration of Helsinki. All data were coded and analyzed anonymously.

No a priori sample size calculation was performed, as this study was conceived as exploratory. The inclusion criteria were children aged 5 to 8 years, with no diagnosed ocular pathology such as strabismus, amblyopia, or cataracts, and no systemic diseases that could affect visual function, including diabetes, neurological disorders, or metabolic diseases. Children were also required not to have undergone any previous visual treatment, such as vision therapy, occlusive exercises, or the use of special lenses, since these could influence visual function or the assessed skills, potentially affecting the validity of the study results.

### 2.2. Procedure

All examinations were conducted in a large school room. To ensure consistency across participants, testing was performed under the same ambient lighting conditions (370 lux). Illuminance levels were measured using a PCE-174 lux meter (PCE Instruments, Meschede, Germany), and lighting was provided by several fixed ceiling spotlights. Four examiners worked simultaneously, positioned approximately two meters apart, with participants rotating between them to complete the full test battery (one hour per child).

Each participant was evaluated using their prescribed optical correction. These assessments were performed by a team of four optometrists, trained to ensure consistency and eliminate potential inter-examiner bias: examiner A performed visual acuity measurements, examiner B conducted phoria assessments (Maddox test), examiner C handled oculomotor evaluations (NSUCO and DEM), and examiner D evaluated the near point of convergence and supervised the CISS V-15.

The specific protocols for these assessments were as follows:

**1. Pre-evaluation preparation.** Parents completed the CISS V-15 a few days prior to the evaluation to assess visual symptoms [8].

**2. Visual acuity measurement. Visual acuity (VA)** was assessed at 5 m for distance vision (with the optotype calibrated for that distance) and 40 cm for near vision using the ETDRS optotype on a LogMAR scale, both monocularly and binocularly. The optotype was presented on a screen and scored letter by letter. The ETDRS test was administered using the OptoTab^®^ app (v. 2.3.0, Smarthings4Vision^®^, Zaragoza, Spain) on a 10-inch Android tablet (BQ Aquaris M10 FHD, Mundo Reader S.L., Las Rozas, Spain) with a screen resolution of 1920 × 1200 pixels [28].

**3. Phoria detection and measurement.** The Maddox test was used to assess the presence of phoria in both distance vision at 5 m (DV) and near vision (NV) at a 40 cm. The Maddox test was selected for assessing phoria because it provides a quick and simple measurement, easier to administer to young children in school settings. The evaluation was performed with a point light stimulus placed on the midline of the eyes and a red filter, with the stripes oriented horizontally and placed on the right eye, recording only the break point. The deviation was quantified using a prism bar, and vertical deviations were not measured [29].

Phoria values were recorded in prism diopters (Δ), specifying both magnitude and direction of the deviation. Exophoria was recorded as negative and esophoria as positive values.

For analytical purposes, both the magnitude of the deviation (absolute value, irrespective of direction) and the direction (exo vs. eso) were considered, depending on the specific analysis performed. This allowed differentiation between the size of the misalignment and its orientation.

**4. Near point of convergence (NPC).** The child was instructed to fixate on a small visual target (Lang bar) held at eye level, which was slowly moved toward the nose bridge. The examiner noted the point at which one eye lost fixation (break point) or the child reported diplopia. The recovery point was not assessed to keep the procedure brief and feasible in the school setting and to minimize fatigue in this age group. The distance from the target to the bridge of the nose at the break point was measured in centimeters [30].

**5. Subjective assessment of saccadic eye movements (NSUCO Test)** [18]. Saccadic and pursuit eye movements were evaluated subjectively using the NSUCO Oculomotor Test, which includes a standardized five-point scoring system with specific criteria.

During saccadic evaluation, each child was positioned 40 cm from two fixation spheres (0.5 cm in diameter) mounted on rods placed 10 cm on either side of the midline. The child was instructed to look alternately at each target. The examiner rated three areas on a five-point scale (see Table 1).

During pursuit evaluation, one of the same fixation targets used in the saccadic test was held approximately 40 cm from the participant and moved smoothly in a circular path at a constant speed. The child was instructed to follow the target with the eyes only, avoiding head and body movements. The examiner rated four areas on a five-point scale: Ability (whether the participant can perform the required smooth tracking), Accuracy (quality and steadiness of pursuit movements), Head movement (extent of head movement during tracking), and Body Movement (presence of compensatory body movements).

**6. The CISS V-15 (Convergence Insufficiency Symptom Survey, version 15)**. The CISS V-15 is a standardized questionnaire designed to assess symptoms related to CI and other binocular vision dysfunctions. The questionnaire consists of 15 items that measure symptoms such as:

- Difficulty maintaining focus while reading or performing near tasks.
- Blurred or double vision during near-vision activities.
- Eye strain or headaches associated with close visual tasks.
- Loss of concentration or a tendency to avoid prolonged reading tasks.

Patients rate the questions on a scale from 0 to 4, where 0 means never experiences the symptom and 4 means always experiences the symptom. The total score was calculated by summing the responses to all 15 items. All responses were provided by parents. Prior to completing the survey, they received the same instructions: to read each question aloud, briefly discuss it with the child, and select the response that best reflected the frequency of the symptom based on both the discussion and their daily observations. The questionnaire was administered in Spanish, using the version available for Spanish-speaking populations [31].

Because the questionnaire has not been validated for pediatric populations aged 5 to 8 years, these results should be interpreted with caution.

**7. Developmental Eye Movement (DEM).** The DEM test was administered to evaluate oculomotor skills. The test consists of three subtests: two vertical number reading sheets (V1 and V2) and one horizontal number reading sheet (AHT). For each subtest, the time taken by the child to complete the task and the number of errors made were recorded. To control for fatigue effects, the order of testing was consistent for all participants: vertical subtests (V1 and V2) first, followed by the horizontal subtest, with adequate rest breaks as needed.

The times recorded for the horizontal subtest were adjusted according to the standard DEM protocol to account for errors. The total vertical time (VT) was calculated as the sum of the times for V1 and V2, and the DEM ratio was computed as the adjusted horizontal time divided by the total vertical time, providing a normalized measure of horizontal saccadic function relative to vertical naming speed.

Children under 6 years of age were excluded from analyses that included the DEM, as they could not reliably recognize or name numbers.

### 2.3. Dysfunction and Symptom Presence Classification

For the proper classification of subjects regarding the presence of different visual dysfunctions and significant visual symptoms, several criteria were adopted based on current scientific literature reference data.

In the Maddox test, used to evaluate phoria, distance vision (5 m) was considered normal for exophoria values between 0 and 2 prism diopters (Δ), while near vision (40 cm) was considered normal for exophoria values between 0 and 6 Δ. Any value outside of these normative values (either the presence of esophoria or higher exophoria values) was considered indicative of potential binocular dysfunction, in accordance with the criteria established by Cacho-Martínez P et al. [32].

Reference norms published by the Northeastern State University College of Optometry were used to classify performance on the NSUCO saccadic and pursuit tests as normal or indicative of oculomotor dysfunction [18]. With regard to the DEM test, dysfunction classification was based on the reference values established by Garzia RP et al. [33].

### 2.4. Data Analysis

Given the exploratory nature of the study, the children were stratified by sex and divided into three age groups: under 6, 6 to 7 and over 7 years old. Descriptive statistics were used to summarize the sample characteristics, and all statistical tests were performed using R Commander (V. 4.4.1). Data normality was assessed using the Shapiro–Wilk test; as distributions were generally non-Gaussian, the Wilcoxon test was used to evaluate inter-group differences by sex.

To explore correlations between optometric parameters, Spearman’s rank correlation coefficient was utilized due to its robustness against outliers and suitability for non-parametric data.

Moreover, multivariable linear regression models were constructed to evaluate the predictive value of clinical variables. Prior to model fitting, multicollinearity was assessed using the Variance Inflation Factor (VIF), excluding variables with a VIF > 5. To minimize redundancy, representative parameters from each functional group were retained based on their clinical relevance and association strength. For each final model, we reported the regression coefficients (β) and the significance of the predictors (*p*-value), along with the adjusted R^2^, and the overall model significance (*p*-value).

Visual dysfunction classifications were further applied to analyze comorbidities and their associations with visual symptoms. Categorical associations were evaluated using Pearson’s chi-square test, or Fisher’s exact test for expected cell frequencies less than 5. Since a definitive cutoff was not applied to the CISS V-15, scores were treated as continuous variables and compared with dichotomous dysfunction variables using the Mann–Whitney U test.

Finally, to account for multiple testing, *p*-values across correlation and association analyses were adjusted using the Benjamini–Hochberg False Discovery Rate (FDR) procedure, with statistical significance defined as an FDR-adjusted *p*-value < 0.05.

## 3. Results

**Figure 1:**
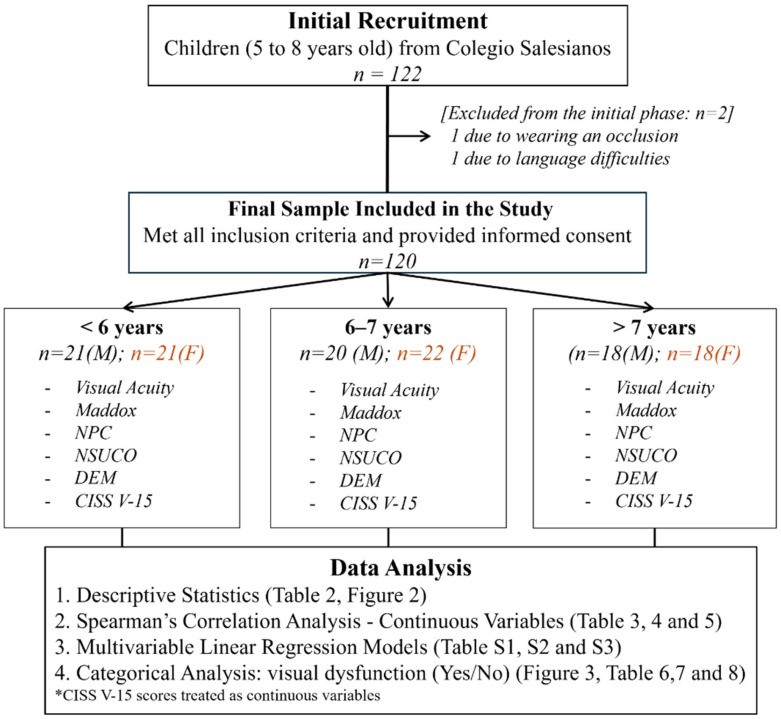
Study flow diagram. The flowchart illustrates the participant selection process, detailing exclusions and the final sample sizes included in each specific analysis. Abbreviations: *n*: number of participants; M: male; F: female; NPC: near point of convergence; NSUCO: subjective assessment of saccadic eye movements; DEM: developmental eye movement; CISS V-15: convergence insufficiency symptom survey, version 15.

The total sample initially consisted of 122 children (Figure 1); however, two participants were excluded (one due to wearing an eye patch and the other due to language difficulties). The final sample comprised 120 children, distributed into three age groups: 42 children under 6 years old (<6 years; 21 males and 21 females), 42 children aged 6 to 7 years (6–7 years; 20 males and 22 females), and 36 children over 7 years old (>7 years; 18 males and 18 females). Children under 6 years of age were not administered the DEM test.

Table 2 presents the binocular descriptive data obtained from the comprehensive optometric examination for the three age groups.

**Figure 2:**
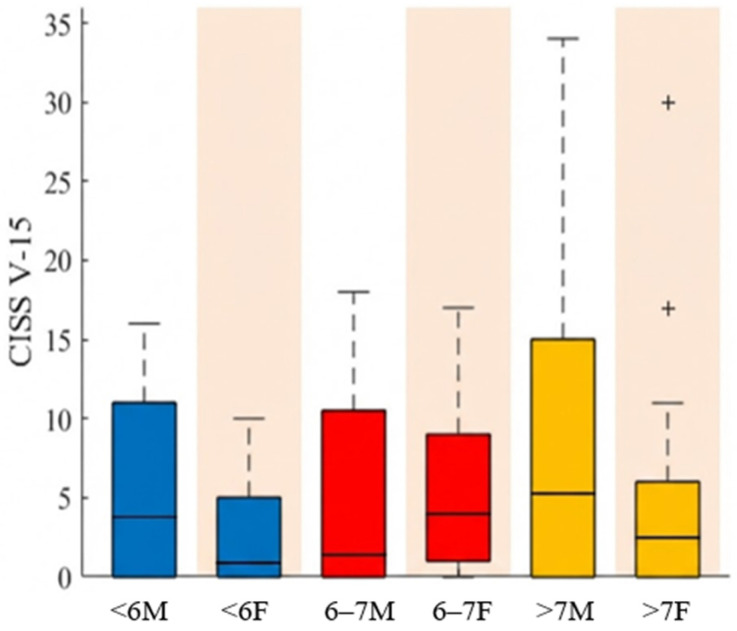
CISS V-15 scores by age group and sex. Age categories are defined as: <6 (younger than 6 years), 6–7 (aged 6 to 7 years), and >7 (older than 7 years). M, male; F, female. (+) represent outliers.

Figure 2 shows the CISS V-15 scores by age and sex. In the <6 age group, males presented a median of 3.8 in a range of [0–16] and females 0.9 [0–10]. Between 6 and 7 years of age, males recorded a median of 1.4 [0–18], while females showed 4.0 [0–17]. In the over-7 age group, males reached the highest median at 5.0 [0–34], and females obtained 2.5 [0–30].

**Figure 3:**
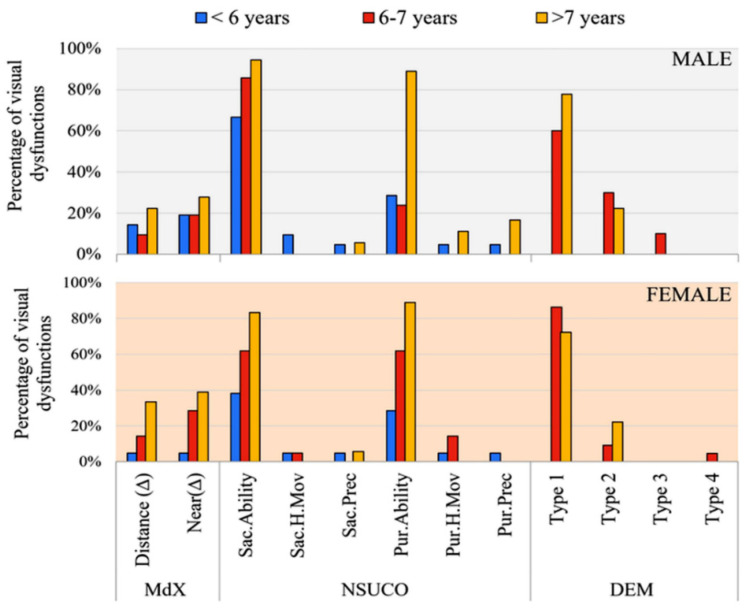
Percentage distribution of children with phorias (Maddox test), and oculomotor dysfunctions (NSUCO and DEM tests), by age group and sex. Dysfunction threshold criteria are detailed in Section 2.3. (Dysfunction and Symptom Presence Classification) of the Methods.

Figure 3 shows the distribution of binocular and oculomotor dysfunctions, which were classified according to the previously published criteria described in the Methods (Section 2.3). A higher proportion of symptomatic children was observed among those older than 7 years presenting with binocular or oculomotor dysfunctions.

Table 3, Table 4 and Table 5 present the results of Spearman’s rank correlations, including the correlation coefficients (r) and *p*-values, among the three age groups evaluated: children under 6 years old (<6 years, Table 3), children between 6 and 7 years old (6–7 years, Table 4), and children over 7 years old (>7 years, Table 5). These tables detail the strength and significance of the associations between visual acuity, oculomotor functions, phorias, and other functional abilities within each group, highlighting both positive and negative correlations depending on the variables analyzed. Positive correlations indicate that as one variable increases, the other tends to increase as well, whereas negative correlations mean that as one variable increases, the other tends to decrease.

In the group of children under 6 years old (Table 3), distance and near phorias showed high correlations, particularly among male participants. Positive correlations were observed between saccadic ability and saccadic accuracy, as well as between pursuit ability and pursuit accuracy. Positive correlations were also found between saccadic movements and pursuit movements. Negative correlations were observed between near phoria and pursuit accuracy and head movements, particularly in girls. The NPC showed negative correlations with both distance and near phorias.

In the group of children aged 6 to 7 years (Table 4), distance and near phorias were positively correlated. Saccadic ability was positively correlated with pursuit ability, and poorer saccadic performance was associated with greater use of compensatory head movements. The DEM test showed a positive correlation with the total vertical time in both boys and girls. No significant correlations were found between the CISS V-15 score and the other variables in this group. No correlations were found with NPC either.

In the group older than 7 years (Table 5), similar patterns were observed. Positive associations were identified between saccadic ability and tracking ability, and between the latter and its accuracy. In girls, the CISS V-15 score correlated positively with near phoria and negatively with NPC. Far phoria was also associated with a higher number of errors and longer execution times on the DEM test.

While the bivariate analyses identified individual associations, multivariable linear regression models were developed to determine the independent contribution of each optometric parameter. The results for the final models predicting CISS V-15, DEM, and NSUCO scores are presented in Tables S1–S3 (Supplementary Materials).

Multivariable regression models revealed that, in the group under 6 years of age, oculomotor skills (NSUCO) exhibited high predictability and internal correlation (R^2^ > 0.80), although symptomatology (CISS V-15) did not show a robust association with clinical variables, particularly in females; in males, distance phoria acted as the primary predictor, whereas age was the determining factor for the development of pursuits in females. Upon reaching the 6–7 year range, a gender-based divergence was observed: the DEM test ratio in females was influenced by distance phoria and age, while in males, the NPC was a significant factor for saccadic precision. Symptoms remained independent of oculomotor efficiency, suggesting that perceived visual fatigue at this age is unrelated to objective clinical findings. Finally, in the group over 7 years of age, the NPC emerged as the critical predictor of symptomatology in both males and females.

Beyond continuous modeling of scores, we further analyzed clinical impact of specific visual dysfunctions by treating them as categorical (dichotomous) variables. Table 6, Table 7 and Table 8 show the results of chi-square tests assessing associations between the presence of phorias and oculomotor dysfunctions, and their relationship with CISS V-15 scores. In addition, Spearman’s rank correlations were calculated to analyze the relationship between the degree of symptoms and oculomotor test scores. DEM results are not shown for children < 6 years old due to the lack of standardized reference values for that age group.

In the under-6 age group (Table 6), we observed statistically significant associations between higher phoria values and higher CISS V-15 scores (*p* < 0.001), as well as between CISS V-15 scores and several NSUCO components (*p* < 0.05). In this group, correlations were also observed between oculomotor skills, especially between accuracy and head movements in saccadic and tracking tasks, reflecting the interdependence between these components and their relation to visual symptoms.

In children aged 6 to 7 years (Table 7), CISS V-15 scores showed some significant associations with distance and near phorias with selected NSUCO parameters. However, these findings were not consistent across all variables and, therefore, are better interpreted as preliminary patterns that may guide future, more targeted studies on symptom–phoria relationships in this age range. Types 3 and 4 of the DEM test were significantly related to variables such as head movements and accuracy in these tasks. Furthermore, correlations were found between head movements and accuracy in saccadic and tracking movements, indicating a complex interaction between these skills and visual symptoms in this group.

In the group older than 7 years (Table 8), the CISS V-15 was significantly associated with near phoria, NPC, and several components of the NSUCO test. Differences were observed in tracking and saccadic values according to different types of visual dysfunctions, with associations between accuracy and head movements in the NSUCO tests. Likewise, types 3 and 4 of the DEM test showed relevant variations in accuracy and head movements during oculomotor tasks.

## 4. Discussion

This study investigated the association between phorias, oculomotor dysfunctions, and visual symptoms in children aged 5 to 8 years. First, descriptive analyses revealed age- and sex-related differences in oculomotor performance and visual symptoms. Oculomotor dysfunctions, particularly those related to saccadic movements, were more frequent in boys than in girls, while tracking impairments did not show significant sex differences. Although performance on the DEM test improved with age in both sexes, boys scored slightly higher than girls. These findings are consistent with previous research. Walker et al. [34] observed differences in oculomotor function between typically developing children and those with sensory processing disorder, suggesting that visual development may be influenced by sex and other individual factors. However, these results should be interpreted with caution, as more studies are needed to explore how neurological development, educational context, and environmental demands influence these differences.

Spearman correlation analyses showed age-dependent patterns in the relationship between oculomotor dysfunctions and visual symptoms. In older children (6 to 7 years and over 7), associations were found between performance on optometric tests and reported symptoms, especially those related to saccadic movements and DEM test results. This could be due to greater symptom awareness or more reliable cooperation during the evaluation. In contrast, in children under 6 years old, correlations between CISS V-15 scores and oculomotor skills were weaker, possibly due to lower symptom awareness or prevalence at that age. This finding is consistent with Menjivar et al. [35], who reported that children over 7 years old with binocular dysfunctions showed more difficulties in saccadic movements and a higher prevalence of symptoms. Similarly, Moiroud et al. [36] highlighted that children with dyslexia exhibit longer fixations and lower performance on the DEM test, emphasizing the importance of evaluating eye movement control during school age. In our sample, DEM performance improved progressively with age, supporting the idea that oculomotor control matures during the early school years.

Across all age groups, significant correlations were observed between distance and near phorias, suggesting that phorias tend to remain stable or change predictably with fixation distance. These results align with the findings of Kim et al. [23], who found more variable fixation in children with phorias. Cantó-Cerdán et al. [37] also reported a trend toward exophoric shift in near vision. Troyer et al. [38] described a similar pattern of increased exophoria as fixation distance decreased, both in children and adults. In relation to this, it is well known that the Maddox test can exhibit variability, especially in the pediatric population [39,40]. This variability can be influenced by factors such as vergence adaptation and inaccurate accommodative response. To minimize vergence adaptation [41], the Maddox test was chosen to be administered first, rather than other tests that involve near vision demands. To minimize potential inaccuracies arising from accommodative fluctuations during the Maddox test, participants were instructed to keep the fixation light as clear as possible throughout the measurement. The different fixation distances were kept constant for all participants and remained stable throughout the entire procedure.

The DEM and NSUCO tests were used for identifying abnormalities in saccadic movements and their association with visual symptoms. Poor performance on these tests was consistently linked to higher CISS V-15 scores. There was a relationship between saccadic and tracking movements: when one was impaired, the other also tended to be affected. Gila L. et al. [42] described similar findings, noting that oculomotor anomalies often occur jointly in tracking, saccadic, and fixation maintenance areas, due to shared neural mechanisms.

In the present study, children with poorer performance on the NSUCO and DEM tests showed higher scores on the CISS V-15, suggesting that even in the absence of ocular disease, subtle oculomotor deficits can impair functional vision. These results align with the conclusions obtained by Croosland et al. [43] and extend them to a pediatric context, highlighting that early detection of oculomotor instability may be crucial to prevent visual fatigue and learning difficulties associated with inefficient binocular control.

For the first time in children aged 5 to 8 years, an association between elevated phoria values and the presence of visual symptoms measured by the CISS V-15 questionnaire is demonstrated, along with clear and statistically significant correlations between oculomotor skills assessed by the NSUCO test and various symptomatic manifestations.

This finding expands current knowledge, indicating that the CISS V-15 questionnaire is not only useful for detecting symptoms related to convergence insufficiency in older populations but also represents a sensitive tool in young children to identify early oculomotor dysfunctions that could affect their visual development and reading skills.

Furthermore, it was revealed, for the first time in this age group, that both saccadic and pursuit movements are significantly related, suggesting a functional interdependence between these two types of eye movements in young children. This interrelationship has been little explored in the literature at early ages and highlights the importance of including both in comprehensive pediatric vision assessments.

However, this study has several limitations. First, although the overall sample was relatively large (*n* = 120), stratification by age and sex created small subgroups of about 20 children each, substantially reducing statistical power and estimate stability. Consequently, while sufficient to identify general patterns, a larger sample would improve reliability and allow for more robust stratified analyses. Second, the regression models may overestimate the strength of associations due to the inclusion of multiple subscales from the same instrument. Additionally, while standardized tools such as DEM and NSUCO were used, future studies should incorporate more objective methods, such as advanced eye-tracking technology, to obtain detailed metrics on the accuracy, latency, and amplitude of eye movements [44,45,46]. This would enhance understanding of oculomotor dysfunctions and their contribution to visual symptoms. The cross-sectional design limits the ability to establish causal relationships or assess long-term effects on visual and academic development.

Moreover, although the CISS-V15 questionnaire has been shown to be a valid tool for assessing visual symptoms in children aged 9 years and older, its application in children younger than 8 years has limited capacity because it relies on parental reports, which can introduce bias or discrepancies. Previous studies have suggested that children’s self-reports can be unreliable due to factors such as limited comprehension of the questions or difficulty identifying specific symptoms. Likewise, it may overestimate symptom severity due to differences in observation or communication with the child. Therefore, results related to visual symptoms and their correlation with oculomotor dysfunctions in this age group should be interpreted with caution [47]. In this context, CISS V-15 scores in our younger participants should be understood as a parental proxy of the child’s symptomatology rather than a direct child self-report and interpreted accordingly in terms of symptom burden.

It is noted that vertical phoria was not assessed in this study, which may overlook subtle binocular misalignments influencing oculomotor performance. Nevertheless, it is also noteworthy to highlight that the use of the Maddox test may represent a limitation of the study, because it does not provide full control over the accommodative response during measurement. A future approach could be the use of the Maddox Wing test [48], including the measurement of vertical deviation.

## 5. Conclusions

The results of this exploratory study showed that the prevalence of dysfunctions in saccadic and pursuit eye movements decreases with age, with sex differences observed only in saccadic movements. Additionally, the proportion of normal results on the DEM test slightly increases with age, especially among girls, for the type I subtest.

Correlations were found between saccadic and pursuit performance, suggesting that both types of eye movements may be related, although we cannot conclude that one causes the other. In the older age group, binocular dysfunctions were associated with poorer saccadic performance and a greater presence of visual symptoms.

The CISS V-15 questionnaire showed that children with binocular vision problems or eye movement difficulties tend to experience more visual symptoms; however, it should be interpreted with caution due to the reliability of visual symptoms in young children.

## Figures and Tables

**Table 1:** Scoring system employed in the NSUCO test for assessing saccadic function.

Performance Area	Evaluation Procedure	Scoring System
Ability	Patient’s ability to perform 5 cycles of fixation change between the two presented stimuli	1 point: 1 cycle or no ability 2 points: 2 cycles 3 points: 3 cycles 4 points: 4 cycles 5 points: 5 cycles
Precision	Patient’s ability to perform 5 cycles of fixation change without corrective refixations	1 point: significant hyper- or hypometric movements 2 points: large to moderate hyper- or hypometric movements 3 points: slight hyper or hypometric movements but constant 4 points: slight hyper or hypometric movements but intermittent 5 points: no correcting refixations
Head movement associated	Patient’s ability to perform 5 cycles of fixation change without head or body movements	1 point: 1 cycle or no ability 2 points: 2 cycles 3 points: 3 cycles 4 points: 4 cycles 5 points: 5 cycles

**Table 2:** Binocular descriptive characteristics of the sample according to age and gender in the evaluated groups: <6 years old, 6–7 years old, and >7 years old, along with the analysis of statistically significant differences between genders. Red italic values indicate statistically significant differences between genders (*p* < 0.05). Orange shading indicates females, and gray shading indicates males.

		<6 Years			6–7 Years			>7 Years		
Gender		Male		Female	Male		Female	Male		Female
Child’s nº		21	*p-val*	21	20	*p-val*	22	18	*p-val*	18
**Age (years)**		5.37 (0.39) [4.90–5.98]	-	5.44 (0.34) [4.94–5.95]	6.40 (0.26) [6.00–6.97]	-	6.41 (0.29) [6.03–6.98]	7.40 (0.23) [7.08–7.79]	-	7.47 (0.32) [7.12–8.20]
**VA (LogMAR)**	**RE**	0.10 (0.07) [0.00–0.20]	*0.18*	0.07 (0.10) [0.00–0.32]	0.04 (0.07) [0.00–0.2]	*0.63*	0.05 (0.08) [0.00–0.30]	0.03 (0.05) [0.00–0.1]	*0.11*	0.08 (0.09) [0.00–0.30]
	**LE**	0.09 (0.09) [0.00–0.30]	*0.11*	0.05 (0.08) [0.00–0.30]	0.04 (0.09) [0.00–0.40]	*0.53*	0.07 (0.10) [0.00–0.30]	0.05 (0.08) [0.00–0.30]	*0.16*	0.09 (0.10) [0.00–0.30]
	**BE**	0.02 (0.04) [0.00–0.10]	*0.71*	0.02 (0.04) [0.00–0.10]	0.01 (0.03) [0.00–0.10]	*0.70*	0.02 (0.07) [0.00–0.3]	0.03 (0.04) [0.00–0.10]	*0.14*	0.06 (0.08) [0.00–0.30]
**MdX**	**Distance**	−1.05 (2.73) [(−10.00)–0.00]	*0.30*	0.29 (1.31) [0.00–6.00]	−0.90 (2.94) [(−12.00)–0.00]	*0.64*	−2.00 (5.14) [(−16.0)–0.00]	−0.78 (3.08) [(−8.00)–6.00]	*0.16*	−2.56 (3.93) [(−12.00)–0.00]
	**Near**	−1.62 (3.56) [(−12.00)–0.0]	*0.04*	0.00 (1.90) [(−6)–6]	−1.70 (3.80) [(−14.00)–0.0]	*0.53*	−3.36 (6.57) [(−20.00)–0.0]	−1.55 (4.63) [(−12.00)–8.00]	*0.35*	−3.44 (5.35) [(−16.00)–4.0]
**NPC (cm)**		3.19 (4.42) [0.00–15.00]	*<0.01*	0.57 (1.43) [0.00–4.00]	3.25 (2.39) [0.00–8.00]	*0.19*	2.36 (2.90) [0.00–8.00]	1.55 (1.98) [0.00–7.00]	*0.04*	3.39 (3.01) [0.00–11.00]
**NSUCO**	**Sac. Ability**	3.86 (1.24) [1.00–5.00]	*0.10*	4.38 (0.92) [2.00–5.00]	3.70 (0.66) [3.00–5.00]	*0.04*	4.09 (1.06) [1.00–5.00]	3.67 (0.68) [2.00–5.00]	*0.27*	3.94 (0.64) [3.00–5.00]
	**Sac. Prec.**	3.90 (1.14) [1.00–5.00]	*0.01*	4.57 (0.50) [2.00–5.00]	3.85 (0.75) [3.00–5.00]	*0.05*	4.23 (0.97) [1.00–5.00]	3.72 (0.67) [3.00–5.00]	*0.15*	3.94 (0.54) [3.00–5.00]
	**Sac. H.Mov**	3.85 (1.11) [1.00–5.00]	*0.07*	4.29 (0.81) [2.00–5.00]	4.05 (0.79) [3.00–5.00]	*0.02*	4.32 (0.72) [3.00–5.00]	3.83 (0.86) [2.00–5.00]	*0.25*	4.22 (0.88) [2.00–5.00]
	**Pur. Ability**	4.05 (1.32) [1.00–5.00]	*0.24*	4.43 (1.06) [1.00–5.00]	3.75 (0.79) [3.00–5.00]	*0.70*	3.91 (1.19) [1.00–5.00]	3.61 (0.98) [1.00–5.00]	*0.92*	3.72 (0.75) [2.00–5.00]
	**Pur. Prec.**	3.95 (1.24) [1.00–5.00]	*<0.01*	4.62 (0.97) [1.00–5.00]	3.86 (1.00) [1.00–5.00]	*0.66*	3.86 (1.21) [1.00–5.00]	3.38 (0.77) [2.00–5.00]	*0.38*	3.56 (0.51) [3.00–4.00]
	**Pur. H.Mov**	4.24 (1.22) [1.00–5.00]	*0.30*	4.52 (0.87) [2.00–5.00]	4.12 (0.77) [3.00–5.00]	*0.05*	4.32 (0.72) [3.00–5.00]	3.66 (0.97) [2.00–5.00]	*0.47*	4.00 (0.91) [3.00–5.00]
	**VT**				63.25 (23.80) [41.22–127.6]	*0.43*	62.36 (14.85) [36.00–99.50]	45.16 (7.59) [33.00–62.00]	*0.69*	44.72 (12.88) [29.00–86.00]
	**AHT**				90.89 (43.87) [43.00–204.0]	*0.85*	85.13 (30.53) [51.00–180.0]	61.38 (26.93) [34.00–140.00]	*0.17*	60.53 (14.38) [42.00–89.00]
	**ERROR**				20.20 (7.78) [10.00–40.00]	*0.20*	16.27 (11.26) [0.00–43.00]	13.50 (9.96) [0.00–33.00]	*0.83*	13.44 (8.08) [0.00–33.00]
	**RATIO**				2.03 (1.09) [1.05–6.09]	*0.56*	1.75 (0.48) [1.12–3.12]	1.66 (0.74) [0.89–4.20]	*0.35*	1.63 (0.46) [0.84–2.67]

Values in parentheses ( ) indicate standard deviation (SD); values in square brackets [ ] indicate range; VA, visual acuity; RE, right eye; LE, left eye; BE, both eyes; NPC, near point of convergence (cm); Mdx, Maddox test; Sac, saccadic; Pur, pursuits; Prec, precision; H.Mov, head movement; VT, total vertical time; AHT, total horizontal time.

**Table 3:** Spearman correlation coefficients (r) and *p*-values FDR between binocular variables in the group of children under 6 years old (<6 years). Red values indicate statistically significant correlations. Orange shading indicates females and gray shading indicates males.

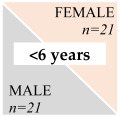		CISS.V-15	Mdx		Age	NSUCO						NPC
			Near	Dist.		Sac. Ability	Sac. H.Mov	Sac. Prec	Pur. Ability	Pur. H.Mov	Pur. Prec	
**CISS V-15**			−0.399 0.048	0.198 0.659	0.093 0.779	−0.322 0.314	−0.212 0.644	−0.402 0.167	−0.517 0.039	−0.467 0.045	−0.498 0.032	−0.104 0.837
**MdX(∆)**	**Near**	0.105 0.746		<0.001 1.000	−0.204 0.644	−0.161 0.683	−0.171 0.683	−0.112 0.767	−0.416 0.152	−0.416 0.152	−0.446 0.048	−0.477 0.125
	**Dist.**	0.105 0.746	0.840 <0.001		0.147 0.700	0.170 0.683	0.185 0.659	0.139 0.700	0.139 0.700	0.139 0.700	0.107 0.767	−0.354 0.779
**Age**		0.091 0.775	0.131 0.396	−0.007 0.583		−0.208 0.644	−0.098 0.779	−0.233 0.606	0.015 0.984	0.126 0.732	0.056 0.854	0.235 0.241
**NSUCO**	**Sac. Ability**	−0.291 0.388	0.406 0.094	0.291 0.468	0.221 0.492		0.920 <0.001	0.873 <0.001	0.884 <0.001	0.861 0.001	0.713 0.005	−0.121 0.641
	**Sac. H.Mov**	−0.231 0.492	0.452 0.049	0.296 0.397	0.160 0.579	0.792 <0.001		0.761 <0.001	0.825 <0.001	0.836 <0.001	0.682 0.024	−0.376 0.659
	**Sac. Prec**	−0.319 0.349	0.457 0.043	0.307 0.388	0.258 0.388	0.843 <0.001	0.931 <0.001		0.826 <0.001	0.757 0.003	0.818 <0.001	−0.315 0.244
	**Pur. Ability**	−0.152 0.652	0.531 0.036	0.500 0.031	0.121 0.622	0.830 <0.001	0.656 0.003	0.681 0.006		0.982 <0.001	0.801 <0.001	0.854 <0.001
	**Pur. H.Mov**	−0.341 0.305	0.543 0.027	0.464 0.047	0.094 0.617	0.612 0.010	0.524 0.054	0.551 0.032	0.794 <0.001		0.771 <0.001	−0.020 1.000
	**Pur. Prec**	−0.083 0.785	0.487 0.032	0.487 0.023	−0.062 0.948	0.682 0.005	0.580 0.028	0.633 0.010	0.846 <0.001	0.835 <0.001		−0.179 0.683
**NPC**		−0.104 0.746	−0.692 0.012	−0.580 0.038	0.200 0.464	−0.212 0.704	−0.404 0.096	−0.352 0.304	−0.098 0.998	−0.110 0.948	−0.218 0.384	

Abbreviations: Mdx, Maddox test; Dist. distance; Sac, saccadic; Pur, pursuits; Prec, precision; H.Mov, head movement; NPC, near point of convergence (cm).

**Table 4:** Spearman correlation coefficients (r) and *p*-values FDR between binocular variables in the group of children aged 6 to 7 years (6–7 years). Red values indicate statistically significant correlations (*p* < 0.05). Orange shading indicates females and gray shading indicates males.

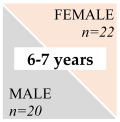		CISS V-15	Mdx		DEM					NSUCO						
			Near	Distance	AHT	ERROR	RATIO	VT	Age	Sac. Ability	Sac. H.Mov	Sac. Prec	Pur. Ability	Pur. H.Mov	Pur. Prec	NPC
	**CISS V-15**		0.309 0.404	0.255 0.463	−0.075 0.498	−0.104 0.287	−0.051 0.324	−0.125 0.486	0.213 0.631	0.040 0.514	0.303 0.892	0.276 0.408	0.146 0.419	0.284 0.631	−0.193 0.419	−0.193 0.537
**Mdx**	**Near**	−0.055 0.899		0.756 <0.001	−0.157 0.532	−0.323 0.947	0.022 0.691	0.009 0.198	0.196 0.643	−0.100 0.537	0.162 0.723	0.247 0.620	0.169 0.474	0.227 0.605	−0.769 0.500	−0.769 <0.001
	**Distance**	−0.065 0.882	0.745 0.003		−0.024 0.504	−0.358 0.093	0.165 0.781	0.187 0.410	0.286 0.514	0.089 0.419	0.239 0.998	0.337 0.474	0.239 0.362	0.314 0.474	−0.572 0.404	−0.571 0.017
	**ATH**	−0.169 0.498	−0.148 0.532	−0.158 0.504		0.381 0.344	−0.307 <0.001	−0.289 0.574	0.140 0.782	0.159 0.900	0.201 0.157	0.111 0.138	−0.035 0.188	0.119 0.526	0.154 0.259	0.154 0.259
**DEM**	**ERROR**	0.252 0.287	0.015 0.947	−0.386 0.093	−0.225 0.344		−0.368 0.477	−0.379 0.239	−0.046 0.990	0.024 0.989	0.057 0.588	−0.039 0.132	−0.136 0.652	−0.085 0.346	0.045 0.851	0.045 0.851
	**RATIO**	−0.235 0.324	0.094 0.691	0.066 0.781	0.791 <0.001	−0.168 0.477		0.977 0.009	−0.259 0.583	−0.195 0.413	−0.396 0.125	−0.269 0.326	−0.187 0.371	−0.225 0.704	0.200 0.121	0.200 0.121
	**VT**	0.165 0.486	0.303 0.198	0.460 0.041	0.133 0.574	0.276 0.239	−0.378 0.099		−0.216 0.613	−0.200 0.689	−0.355 0.911	−0.244 0.943	−0.180 0.561	−0.192 0.220	0.229 0.210	0.229 0.210
**Age**		0.310 0.355	0.066 0.882	0.153 0.733	−0.066 0.782	0.990 <0.001	−0.138 0.583	0.006 0.980		0.789 0.034	0.533 0.043	0.592 0.054	0.387 0.710	0.407 0.027	0.385 0.854	0.001 0.998
**NSUCO**	**Sac. Ability**	−0.253 0.452	0.326 0.355	0.081 0.882	−0.030 0.900	0.131 0.573	−0.030 0.900	0.085 0.721	−0.179 0.920		0.432 <0.001	0.457 <0.001	0.281 <0.001	0.348 <0.001	0.247 <0.001	−0.207 0.514
	**Sac. H.Mov**	0.087 0.882	0.228 0.508	0.342 0.355	−0.329 0.157	−0.129 0.588	−0.351 0.125	0.026 0.911	−0.153 0.882	0.608 0.034		0.788 0.002	0.601 0.001	0.618 <0.001	0.276 0.001	−0.152 0.631
	**Sac. Prec**	0.317 0.355	0.250 0.452	−0.021 0.920	−0.367 0.111	0.343 0.138	−0.387 0.096	−0.017 0.943	0.125 0.869	0.417 0.314	0.303 0.355		0.874 <0.001	0.940 <0.001	0.317 <0.001	−0.208 0.514
	**Pur. Ability**	−0.333 0.355	0.324 0.355	0.211 0.551	−0.307 0.188	−0.063 0.801	0.047 0.842	−0.596 0.006	0.205 0.433	0.501 0.047	0.390 0.355	0.378 0.341		0.935 <0.001	0.356 <0.001	−0.294 0.419
	**Pur. H.Mov**	0.091 0.882	0.307 0.355	0.400 0.314	−0.150 0.526	−0.222 0.346	−0.090 0.704	−0.287 0.220	0.049 0.882	0.485 0.031	0.529 0.001	0.153 0.314	0.302 0.018		−0.285 <0.001	−0.328 0.374
	**Pur. Prec**	−0.280 0.410	0.389 0.314	0.143 0.752	−0.116 0.624	0.001 0.996	0.164 0.488	−0.487 0.032	0.252 0.433	0.598 0.028	0.477 0.355	0.246 0.314	0.529 <0.001	0.405 0.018		−0.251 0.463
**NPC**		0.031 0.929	−0.199 0.577	−0.034 0.920	−0.295 0.206	0.236 0.315	−0.335 0.147	0.083 0.726	0.107 0.920	0.002 0.314	0.034 0.410	−0.237 0.869	0.048 0.355	0.129 0.355	0.089 0.230	

Abbreviations: Mdx, Maddox test; VT, total vertical time; AHT, horizontal time; Sac, saccadic; Pur, pursuits; Prec, precision; H.Mov, head movement; NPC, near point of convergence (cm).

**Table 5:** Spearman correlation coefficients (r) and *p*-values FDR between binocular variables in the group of children over 7 years old (>7 years). Red values indicate statistically significant correlations (*p* < 0.05). Orange shading indicates females, and gray shading indicates males.

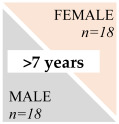		CISS.V-15	Mdx			DEM			Age	NSUCO						NPC
			Near	Distance	AHT	ERROR	RATIO	VT		Sac. Ability	Sac. H.Mov	Sac. Prec	Pur. Ability	Pur. H.Mov	Pur. Prec	
	**CISS. V-15**		0.902 0.012	−0.302 0.803	−0.257 0.552	−0.075 0.498	−0.149 0.672	−0.247 0.267	−0.074 0.642	0.079 0.659	0.156 0.761	0.444 0.821	0.329 0.729	0.397 0.909	0.492 0.607	0.453 0.232
**Mdx**	**Near**	−0.162 0.897		−0.444 <0.001	−0.557 0.532	−0.037 0.987	−0.165 0.245	−0.400 0.002	−0.183 0.664	0.088 0.729	0.073 0.659	0.323 0.168	0.365 0.296	0.351 0.245	0.546 0.032	−0.625 0.061
	**Distance**	−0.305 0.706	0.920 <0.001		−0.024 0.504	0.534 <0.001	0.278 0.665	0.523 <0.001	0.086 0.542	0.040 0.659	−0.135 0.803	−0.347 0.296	−0.356 0.267	−0.098 0.296	−0.171 0.080	−0.678 0.036
	**AHT**	−0.269 0.598	−0.348 0.532	−0.558 0.504		0.269 0.322	−0.207 0.326	−0.389 0.374	0.240 0.681	0.160 0.801	0.203 0.365	0.121 0.238	−0.065 0.188	0.419 0.526	0.554 0.259	0.303 0.465
**DEM**	**ERROR**	0.149 0.498	−0.111 0.532	−0.008 0.504	−0.225 0.344		0.717 <0.001	−0.285 0.325	0.176 0.722	−0.040 0.785	0.074 0.888	−0.124 0.811	−0.069 0.777	0.238 0.824	−0.121 0.834	−0.221 0.624
	**RATIO**	−0.149 0.287	0.327 0.947	0.307 0.093	0.555 <0.001	0.407 0.001		−0.014 0.921	0.113 0.832	−0.031 0.973	0.005 0.999	−0.103 0.721	0.048 0.999	−0.005 0.889	−0.379 0.045	−0.463 0.426
	**VT**	−0.385 0.324	0.014 0.691	0.140 0.781	0.133 0.574	0.162 0.654	−0.055 0.477		−0.171 0.654	0.162 0.654	−0.089 0.999	−0.186 0.538	−0.229 0.125	−0.295 0.095	−0.220 0.416	−0.110 0.526
**Age**		−0.068 0.939	−0.158 0.897	−0.306 0.706	0.233 0.102	−0.066 0.782	−0.306 0.176	−0.158 0.864		0.141 0.920	−0.139 0.659	<0.001 0.659	−0.144 0.558	0.059 0.886	0.098 0.932	0.251 0.543
**NSUCO**	**Sac. Ability**	0.213 0.869	0.043 0.952	−0.055 0.949	−0.030 0.900	−0.256 0.157	0.020 0.999	−0.229 0.088	−0.129 0.909		0.153 0.659	0.408 0.023	0.513 0.015	0.345 0.026	0.604 0.042	−0.280 0.511
	**Sac. H.Mov**	−0.116 0.909	0.087 0.909	0.102 0.909	−0.329 0.157	−0.498 <0.001	−0.241 0.588	−0.190 0.583	0.205 0.869	0.292 0.719		0.335 0.327	0.233 0.542	0.687 0.036	0.277 0.886	0.255 0.543
	**Sac. Prec**	0.218 0.869	−0.176 0.897	−0.279 0.719	−0.367 0.111	−0.081 0.999	−0.021 0.456	−0.363 0.125	0.156 0.897	0.696 0.014	0.447 0.266		0.604 0.027	0.277 0.327	0.246 0.334	0.365 0.327
	**Pur. Ability**	0.426 0.306	−0.119 0.909	−0.263 0.762	−0.307 0.188	−0.308 0.234	0.062 0.996	−0.370 0.145	0.103 0.909	0.803 0.001	0.242 0.838	0.683 0.014		0.320 0.327	0.551 0.080	−0.317 0.408
	**Pur. H.Mov**	0.121 0.909	−0.096 0.909	−0.054 0.296	−0.150 0.949	−0.248 0.564	−0.096 0.874	−0.234 0.704	−0.099 0.909	0.764 0.003	0.446 0.266	0.681 0.0147	0.662 0.018		0.469 0.103	−0.098 0.802
	**Pur. Prec**	0.280 0.717	−0.028 0.959	−0.162 0.897	−0.116 0.624	−0.249 0.256	0.087 0.873	−0.278 0.488	0.179 0.897	0.613 0.036	0.232 0.842	0.641 0.025	0.794 0.001	0.540 0.013		−0.602 0.075
**NPC**		0.367 0.488	0.018 0.959	−0.086 0.909	−0.246 0.237	−0.295 0.206	0.059 0.785	−0.369 0.147	−0.021 0.959	−0.112 0.909	−0.042 0.952	0.164 0.897	0.016 0.959	0.017 0966	0.440 0.009	

Abbreviations: Mdx, Maddox test; VT, total vertical time; AHT, horizontal time; Sac, saccadic; Pur, pursuits; Prec, precision; H.Mov, head movement; NPC, near point of convergence (cm).

**Table 6:** Associations between different oculomotor dysfunctions, and between oculomotor dysfunctions, in children under 6 years of age (<6 years). Statistically significant associations (*p*-values FDR < 0.05) are shown in red. “NA” indicates variables with no variability, which prevented chi-square analysis. Orange shading indicates females, and gray shading indicates males.

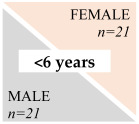			Mdx		NSUCO					
		CISS V-15	Near	Distance	Sac. Ability	Sac. H.Mov	Sac. Prec	Pur. Ability	Pur. H.Mov	Pur. Prec
**CISS V-15**			<0.001	<0.001	0.027	<0.001	<0.001	0.049	<0.001	<0.001
**Mdx**	**Near**	0.032		0.818	0.191	0.667	0.818	0.516	0.818	0.518
	**Distance**	<0.001	<0.001		0.421	0.728	0.645	0.319	0.736	0.641
**NSUCO**	**Sac. Ability**	0.126	0.116	0.185		0.191	0.191	<0.001	0.191	0.191
	**Sac. H.Mov**	<0.001	0.675	0.674	0.458		<0.001	0.045	<0.001	<0.001
	**Sac. Prec**	0.031	0.243	0.129	0.293	<0.001		0.045	<0.001	<0.001
	**Pur. Ability**	0.049	0.291	0.112	0.041	0.042	0.018		0.045	0.045
	**Pur. H.Mov**	<0.001	0.619	0.629	0.468	0.001	<0.001	0.105		<0.001
	**Pur. Prec**	0.012	0.615	0.619	0.468	0.001	<0.001	0.092	<0.001	

Abbreviations: Mdx, Maddox test; Sac, saccades; Pur, pursuits; Prec, precision; H.Mov, head movements. Dysfunction threshold criteria are detailed in Section 2.3. (Dysfunction and Symptom Presence Classification) of the Methods.

**Table 7:** Associations between different oculomotor dysfunctions, and between oculomotor dysfunctions, in children aged 6 to 7 years. Statistically significant associations (*p*-values *FDR* < 0.05) are shown in red. “NA” indicates variables with no variability, preventing chi-square analysis. Orange shading indicates females and gray shading indicates males.

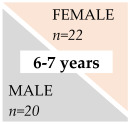		CISS V-15	Mdx		NSUCO						DEM			
			Near	Distance	Sac. Ability	Sac. H.Mov	Sac. Prec	Pur. Ability	Pur. H.Mov	Pur. Prec	Type 1	Type 2	Type 3	Type 4
**CISS V-15**			0.532	0.684	0.039	<0.001	<0.001	0.023	0.039	<0.001	0.684	0.684	0.746	0.568
**Mdx**	**Near**	0.264		0.002	0.658	0.033	0.531	0.658	0.033	0.099	0.099	0.015	0.033	0.531
	**Distance**	0.047	0.003		0.121	0.001	0.684	0.121	0.001	0.285	0.285	0.116	0.001	0.684
**NSUCO**	**Sac. Ability**	0.041	0.456	0.619		0.394	0.394	<0.001	0.394	0.121	0.774	0.784	0.394	0.394
	**Sac. H.Mov**	<0.001	0.021	<0.001	<0.001		<0.001	0.394	NA	0.001	<0.001	<0.001	NA	<0.001
	**Sac. Prec**	<0.001	0.005	<0.001	<0.001	NA		0.394	<0.001	0.01	0.01	0.746	<0.001	<0.001
	**Pur. Ability**	0.001	0.197	0.389	0.038	0.025	0.025		0.394	0.121	0.774	0.784	0.394	0.394
	**Pur. H.Mov**	<0.001	0.003	<0.001	<0.001	NA	NA	0.025		0.001	<0.001	<0.001	NA	<0.001
	**Pur. Prec**	<0.001	0.015	<0.001	<0.001	NA	NA	0.025	NA		0.285	0.556	<0.001	<0.001
**DEM**	**Type 1**	0.761	0.648	0.068	0.224	0.371	0.371	0.291	0.371	0.371				
	**Type 2**	0.389	0.875	0.389	0.389	0.025	0.253	0.765	0.025	0.025				
	**Type 3**	0.144	0.531	0.144	0.531	0.001	0.001	0.072	0.001	0.002				
	**Type 4**	0.732	0.608	0.732	0.732	<0.001	<0.001	0.053	<0.001	<0.001				

Abbreviations: Mdx, Maddox test; Sac, saccades; Pur, pursuits; Prec, precision; H.Mov, head movements; DEM Type 1, normal; DEM Type 2, oculomotor dysfunction; DEM Type 3, automated dysfunction; DEM Type 4, oculomotor + automated dysfunction. Dysfunction threshold criteria are detailed in Section 2.3. (Dysfunction and Symptom Presence Classification) of the Methods.

**Table 8:** Associations between different oculomotor dysfunctions and between oculomotor dysfunctions in children older than 7 years. Statistically significant associations (*p*-values *FDR* < 0.05) are shown in red. “NA” indicates variables with no variability, preventing chi-square analysis. Orange shading indicates females and gray shading indicates males.

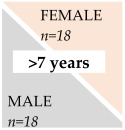		CISS V-15	Mdx		NSUCO						DEM			
			Near	Distance	Sac. Ability	Sac. H.Mov	Sac. Prec	Pur. Ability	Pur. H.Mov	Pur. Prec	Type 1	Type 2	Type 3	Type 4
**CISS V-15**			0.326	0.421	0.732	0.645	0.621	0.547	0.429	0.332	0.847	0.612	0.807	0.531
**Mdx**	**Near**	0.239		<0.001	0.829	0.197	0.346	0.582	0.346	0.456	0.255	0.605	0.346	0.346
	**Distance**	0.311	<0.001		0.847	0.467	0.157	0.596	0.227	0.157	0.423	0.157	0.157	0.157
**NSUCO**	**Sac. Ability**	0.645	0.097	0.054		0.645	0.004	0.179	0.004	0.004	0.239	0.312	0.004	0.004
	**Sac. H.Mov**	0.645	0.523	0.582	0.803		<0.001	0.716	<0.001	<0.001	0.523	0.582	<0.001	<0.001
	**Sac. Prec**	0.005	0.059	0.018	<0.001	<0.001		<0.001	NA	NA	0.059	0.018	NA	NA
	**Pur. Ability**	0.502	0.457	0.316	0.004	0.716	0.001		<0.001	0.009	0.352	0.422	<0.001	0.010
	**Pur. H.Mov**	0.396	0.239	0.311	0.645	0.021	0.005	0.502		NA	0.059	0.018	NA	NA
	**Pur. Prec**	0.502	0.457	0.423	0.716	0.716	0.001	0.597	0.502		0.059	0.018	NA	NA
**DEM**	**Type 1**	0.311	0.758	0.632	0.412	0.582	0.018	0.267	0.532	0.422				
	**Type 2**	0.401	0.648	0.882	0.582	0.447	<0.001	0.442	0.612	0.326				
	**Type 3**	0.023	0.042	<0.001	<0.001	<0.001	NA	<0.001	0.004	<0.001				
	**Type 4**	0.005	0.039	0.018	<0.001	<0.001	NA	<0.001	<0.001	<0.001				

Abbreviations: Mdx, Maddox test; Sac, saccades; Pur, pursuits; Prec, precision; H.Mov, head movements; DEM Type 1, normal; DEM Type 2, oculomotor dysfunction; DEM Type 3, automated dysfunction; DEM Type 4, oculomotor + automated dysfunction. Dysfunction threshold criteria are detailed in Section 2.3. (Dysfunction and Symptom Presence Classification) of the Methods.

## Data Availability

Data is contained within the article or Supplementary Materials.

## Supplementary Materials

The following supporting information can be downloaded at https://www.mdpi.com/article/10.3390/jemr19020036/s1. Table S1: Multivariable linear regression models for clinical predictors of visual performance and symptoms in children under 6 years of age (males and females). Table S2: Multivariable linear regression models for clinical predictors of visual performance and symptoms in children aged 6–7 years (males and females). Table S3: Multivariable linear regression models for clinical predictors of visual performance and symptoms in children aged >7 years (males and females).
