# Supplementary material for: An Exploratory Study of the Relationship Between Phoria, Oculomotor Skills and Visual Symptoms in Children Aged 5 to 8 Years
Source: J Eye Mov Res. 2026 Apr 2;19(2):36. doi: 10.3390/jemr19020036 (PMC13116892; doi:10.3390/jemr19020036)
Supplement: Supplementary file 1 [file jemr-19-00036-s001.zip › jemr-4128632-supplementary.pdf]

Journal of *Eye Movement Research*

MDPI

Supplementary Materials

An Exploratory Study of the Relationship Between Phoria, Oculomotor Skills and Visual Symptoms in Children Aged 5 to 8 Years

Carmen Bilbao [1,2,3,*], Julia Cavero [1], Jorge Ares [2], Alba Carrera [1,2] and Diana Gargallo Yebra[2]

1 Department of Optometry, Hospital Quirón, Huesca, Spain
2 Department of Applied Physics, Universidad de Zaragoza, 50009, Spain
3 Hospital Universitario Miguel Servet, Zaragoza, Spain
* Correspondence: carmenbilbao@unizar.es

Table S1. Multivariable linear regression models for clinical predictors of visual performance and symptoms in children under 6 years of age (males and females). Cells show regression coefficients (β) with p-values; the final column reports the adjusted R^2 and the model p-value. Red values indicate statistically significant correlations. Orange shading indicates females and gray shading indicates males.

< 6 years β (p-value)		PREDICTORS												
			Mdx			NSUCO								
		CISS V-15	Near	Distance	Age	Sac. Ability	Sac. H.Mov	Sac. Prec	Pur. Ability	Pur. H.Mov	Pur. Prec	NPC	R^2 Adj. p-value	
	CISS V-15		0.051<br>0.907	-1.070<br>0.017	3.280<br>0.361			-2.281<br>0.040				-0.613<br>0.142	0.255<br>0.089	MALE (n=21)
NSUCO	Sac. Abilit		-0.103<br>0.025	-0.016<br>0.669	0.513<br>0.134				0.894<br><0.001			-0.072<br>0.066	0.864<br><0.001	
	Sac. H.Mov	-0.005<br>0.835	0.003<br>0.872	-0.011<br>0.565	-0.146<br>0.406			0.964<br><0.001				-0.005<br>0.815	0.955<br><0.001	
	Sac. Prec		-0.005<br>0.835	0.011<br>0.600	0.181<br>0.309		0.997<br><0.001					0.001<br>0.980	0.956<br><0.001	
	Pur. Ability		0.113<br>0.019	0.017<br>0.674	-0.441<br>0.227	0.990<br><0.001						0.071<br>0.091	0.868<br><0.001	
	Pur. H.Mov		0.009<br>0.847	0.017<br>0.698	0.003<br>0.994						0.923<br><0.001	0.029<br>0.486	0.827<br><0.001	
	Pur. Prec		0.004<br>0.931	-0.020<br>0.644	0.019<br>0.959					0.928<br><0.001		-0.033<br>0.431	0.833<br><0.001	
	CISS V-15			0.266<br>0.631	1.076<br>0.634				1.191<br>0.085			-0.001<br>0.998	0.007<br>0.419	FEMALE (n=21)
NSUCO	Sac. Abilit			0.034<br>0.547	-0.285<br>0.222				0.787<br><0.001			-0.064<br>0.237	0.885<br><0.001	
	Sac. H.Mov			0.042<br>0.548	-0.541<br>0.070					1.141<br><0.001		-0.085<br>0.205	0.867<br><0.001	
	Sac. Prec			0.030<br>0.558	-0.321<br>0.136						0.741<br><0.001	-0.077<br>0.125	0.877<br><0.001	
	Pur. Ability			-0.027<br>0.686	0.294<br>0.300	1.143<br><0.001						0.074<br>0.258	0.878<br><0.001	
	Pur. H.Mov			-0.014<br>0.804	0.591<br>0.016				0.764<br><0.001			0.015<br>0.765	0.878<br><0.001	
	Pur. Prec			-0.028<br>0.668	0.358<br>0.195			1.197<br><0.001				0.086<br>0.184	0.862<br><0.001	

Abbreviations: Mdx, Maddox test; Dist., distance; VT, total vertical time; AHT, horizontal time; Sac, saccadic; Pur, pursuits; Prec, precision; H.Mov, head movement; NPC, near point of convergence (cm).

Table S2. Multivariable linear regression models for clinical predictors of visual performance and symptoms in children aged 6–7 years (males and females). Cells show regression coefficients (β) with p-values; the final column reports the adjusted R^2 and the model p-value. Red values indicate statistically significant correlations. Orange shading indicates females and gray shading indicates males.

6-7 years		β (p-value)	CISS V-15	Mdx Near	Mdx Dist.	DEM AHT	DEM ERROR	DEM RATIO	DEM VT	Age	NSUCO Sac. Abilit	NSUCO Sac. H.Mov	NSUCO Sac. Prec	NSUCO Pur. Ability	NSUCO Pur. H.Mov	NSUCO Pur. Prec	NPC	R^2 Adj. p-value
MALE (n=20)		CISS V-15					0.299<br>0.153			3.102<br>0.589							0.385<br>0.562	0.069<br>0.260
MALE (n=20)	DEM	AHT					-1.793<br>0.000	35.435<br>0.000	1.314<br>0.000	-7.896<br>0.424			-3.415<br>0.340				-1.752<br>0.148	0.948<br>0.000
MALE (n=20)	DEM	ERROR							0.103<br>0.195	4.950<br>0.471			3.262<br>0.178				0.570<br>0.470	0.151<br>0.173
MALE (n=20)	DEM	RATIO				0.016<br>0.002	-0.001<br>0.962			1.112<br>0.142		-0.432<br>0.146	0.295<br>0.352				-0.124<br>0.153	0.536<br>0.010
MALE (n=20)	DEM	VT					0.500<br>0.382			-26.28<br>0.096			12.189<br>0.075	-27.89<br>0.001			0.167<br>0.926	0.557<br>0.004
MALE (n=20)	NSUCO	Sac. Ability								-0.036<br>0.944		0.429<br>0.021					-0.075<br>0.192	0.273<br>0.044
MALE (n=20)	NSUCO	Sac. H.Mov								0.065<br>0.888					0.812<br>0.000		0.011<br>0.829	0.588<br>0.001
MALE (n=20)	NSUCO	Sac. Prec								-0.614<br>0.297	0.687<br>0.010						0.142<br>0.046	0.283<br>0.040
MALE (n=20)	NSUCO	Pur. Ability								-0.074<br>0.800	0.099<br>0.468					0.821<br>0.000	0.028<br>0.418	0.798<br>0.000
MALE (n=20)	NSUCO	Pur. H.Mov								0.058<br>0.901	-0.073<br>0.754	0.816<br>0.000					-0.047<br>0.393	0.580<br>0.002
MALE (n=20)	NSUCO	Pur. Prec								-0.076<br>0.797				0.934<br>0.000			-0.044<br>0.183	0.821<br>0.000
FEMALE (n=22)		CISS V-15								0.919<br>0.815						1.239<br>0.205		-0.008<br>0.416
FEMALE (n=22)	DEM	AHT					-1.258<br>0.000	47.460<br>0.000	1.255<br>0.000	-2.027<br>0.748			-2.549<br>0.238					0.941<br>0.000
FEMALE (n=22)	DEM	ERROR						8.757<br>0.081		-10.88<br>0.176								0.142<br>0.091
FEMALE (n=22)	DEM	RATIO			-0.017<br>0.009	0.017<br>0.000	0.026<br>0.000		-0.021<br>0.000	0.219<br>0.048		0.064<br>0.114						0.942<br>0.000
FEMALE (n=22)	DEM	VT				0.291<br>0.004				-11.92<br>0.207		-1.923<br>0.625						0.369<br>0.010
FEMALE (n=22)	NSUCO	Sac. Ability								-0.065<br>0.816				0.847<br>0.000				0.882<br>0.000
FEMALE (n=22)	NSUCO	Sac. H.Mov								-0.400<br>0.216					0.799<br>0.000			0.657<br>0.000
FEMALE (n=22)	NSUCO	Sac. Prec								0.245<br>0.330	0.854<br>0.000							0.887<br>0.000
FEMALE (n=22)	NSUCO	Pur. Ability								0.325<br>0.039						0.966<br>0.000		0.973<br>0.000
FEMALE (n=22)	NSUCO	Pur. H.Mov								-0.498<br>0.101	0.579<br>0.000							0.710<br>0.000
FEMALE (n=22)	NSUCO	Pur. Prec								-0.323<br>0.048				1.000<br>0.000				0.972<br>0.000

Abbreviations: Mdx, Maddox test; Dist., distance; VT, total vertical time; AHT, horizontal time; Sac, saccadic; Pur, pursuits; Prec, precision; H.Mov, head movement; NPC, near point of convergence (cm).

Table S3. Multivariable linear regression models for clinical predictors of visual performance and symptoms in children aged >7 years (males and females). Cells show regression coefficients (β) with p-values; the final column reports the adjusted R² and the model p-value. Red values indicate statistically significant correlations. Orange shading indicates females and gray shading indicates males.

> 7 years			PREDICTORS															
				Mdx		DEM					NSUCO							
		β (p-value)	CISS V-15	Near	Dist.	AHT	ERROR	RATIO	VT	Age	Sac. Abilit	Sac. H.Mov	Sac. Prec	Pur. Abil	Pur. H.Mov	Pur. Prec	NPC	R^2 Adj. p-value
MALE (n=18)		CISS V-15								-6.965<br>0.428		-1.454<br>0.542					2.207<br>**0.047**	0.285<br>0.078
MALE (n=18)	DEM	AHT																
MALE (n=18)	DEM	ERROR																
MALE (n=18)	DEM	RATIO																
MALE (n=18)	DEM	VT					0.045<br>0.834			-3.602<br>0.681		-1.907<br>0.502			-2.415<br>0.294		-1.344<br>0.180	0.068<br>0.347
MALE (n=18)	NSUCO	Sac. Abilit								-0.597<br>0.155		-0.073<br>0.571	0.282<br>0.187	0.615<br>**0.003**	0.173<br>0.204	-0.268<br>0.237	-0.082<br>0.120	0.788<br>**0.002**
MALE (n=18)	NSUCO	Sac. H.Mov								1.097<br>0.225			0.102<br>0.803		0.415<br>0.148		-0.063<br>0.530	0.184<br>0.161
MALE (n=18)	NSUCO	Sac. Prec								0.199<br>0.721		0.205<br>0.192				0.335<br>0.146	0.081<br>0.189	0.514<br>**0.014**
MALE (n=18)	NSUCO	Pur. Ability								0.769<br>0.137	1.040<br>**0.002**	0.102<br>0.504	-0.326<br>0.220		-0.120<br>0.485	0.632<br>**0.009**	0.115<br>0.054	0.841<br>**0.000**
MALE (n=18)	NSUCO	Pur. H.Mov								-0.620<br>0.423	0.845<br>**0.006**	0.428<br>0.067					0.061<br>0.486	0.510<br>**0.009**
MALE (n=18)	NSUCO	Pur. Prec								0.012<br>0.983			0.408<br>0.139		-0.154<br>0.371		-0.085<br>0.169	0.657<br>**0.004**
FEMALE (n=18)		CISS V-15															1.635<br>**0.004**	0.361<br>**0.005**
FEMALE (n=18)	DEM	AHT			-1.898<br>**0.031**													0.223<br>**0.031**
FEMALE (n=18)	DEM	ERROR						11.93<br>**<0.001**										0.432<br>**0.002**
FEMALE (n=18)	DEM	RATIO				0.022<br>**<0.001**	0.026<br>**<0.001**		-0.024<br>**<0.001**									0.920<br>**<0.001**
FEMALE (n=18)	DEM	VT				0.405<br>0.069				-10.59<br>0.365								0.148<br>0.127
FEMALE (n=18)	NSUCO	Sac. Abilit								0.048<br>0.903			0.394<br>0.130			0.667<br>**0.018**		0.398<br>**0.017**
FEMALE (n=18)	NSUCO	Sac. H.Mov								-0.068<br>0.902			0.289<br>0.423			0.575<br>**0.013**		0.358<br>**0.026**
FEMALE (n=18)	NSUCO	Sac. Prec		0.042<br>0.062						-0.188<br>0.589	0.329<br>0.074							0.345<br>**0.030**
FEMALE (n=18)	NSUCO	Pur. Ability								-0.588<br>0.255	0.589<br>0.053		0.089<br>0.798					0.281<br>0.055
FEMALE (n=18)	NSUCO	Pur. H.Mov								0.034<br>0.936		0.754<br>**<0.001**	-0.196<br>0.513			1.010<br>**0.003**		0.645<br>**0.001**
FEMALE (n=18)	NSUCO	Pur. Prec									0.512<br>**0.004**							0.372<br>**0.004**

Abbreviations: Mdx, Maddox test; Dist., distance; VT, total vertical time; AHT, horizontal time; Sac, saccadic; Pur, pursuits; Prec, precision; H.Mov, head movement; NPC, near point of convergence (cm).

Disclaimer/Publisher's Note: The statements, opinions and data contained in all publications are solely those of the individual author(s) and contributor(s) and not of MDPI and/or the editor(s). MDPI and/or the editor(s) disclaim responsibility for any injury to people or property resulting from any ideas, methods, instructions or products referred to in the content.
